# The Emergence of Subclones Following Initial Chemotherapy in Mixed Phenotype Acute Leukemia

**DOI:** 10.7759/cureus.87163

**Published:** 2025-07-02

**Authors:** Taichi Murao, Yusuke Yamane, Miki Kiyota, Naoko Shiozaki, Katsuya Wada, Yuri Kamitsuji, Junya Kuroda, Eri Kawata

**Affiliations:** 1 Hematology, Panasonic Health Insurance Organization Matsushita Memorial Hospital, Osaka, JPN; 2 Clinical Laboratory, Panasonic Health Insurance Organization Matsushita Memorial Hospital, Osaka, JPN; 3 Hematology and Oncology, Kyoto Prefectural University of Medicine, Kyoto, JPN; 4 Hematology, Japanese Red Cross Kyoto Daini Hospital, Kyoto, JPN

**Keywords:** allogeneic hematopoietic stem cell transplantation, lineage-matched therapy, lineage switches, mixed-phenotype acute leukemia, molecular residual disease, myeloperoxidase, subclones

## Abstract

Mixed phenotype acute leukemia (MPAL) is a rare subtype of acute leukemias, and it is characterized by the immunophenotypic expression of multiple hematopoietic lineages and the potential for lineage switching post-treatment. Diagnosing MPAL can be challenging, particularly in cases with small immunophenotypically distinct subclones or subpopulations exhibiting weak antigen expression. We present two cases of MPAL where subclones or lineage switches emerged following initial treatment. Both cases exhibited a myeloperoxidase (MPO)-positive population of leukemic cells, which was initially either undervalued or overestimated, complicating their diagnosis. In one case, leukemic blasts tested negative for MPO in immunohistochemical (IHC) staining; however, flow cytometric analysis revealed a minor subclone that was weakly positive for MPO. In the other patient, leukemic blasts tested positive for MPO through IHC staining. The latter patient was diagnosed with acute myelogenous leukemia; however, the leukemic cells exhibited lymphoblastic morphological features and co-expressed both myeloid and lymphoblastic antigens in each case. Following initial treatments, selective pressure causes the proliferation of leukemic cells resistant to chemotherapy, with an immunophenotypic shift in the blasts, requiring treatment modification in both cases. These two cases highlight the diagnostic and therapeutic complexities of MPAL and underscore the crucial role of comprehensive immunophenotyping in identifying minor subclones, thereby ensuring an accurate diagnosis, informing treatment selection, and facilitating timely therapeutic adjustments.

## Introduction

Mixed phenotype acute leukemia (MPAL) is a type of acute leukemia where blasts display characteristics from multiple hematopoietic cell lineages [1]. MPAL may present as a single blast population with two or more lineage-defining markers (biphenotypic) or as two blast populations representing different lineages (bilinial or bilineage). The latest edition (5th) of the WHO Classification of Hematolymphoid Tumors combines acute leukemia of ambiguous lineage (ALAL) and MPAL into a single entity, categorizing them into two subtypes: one based on genetic abnormalities and the other on immunophenotypes [2]. Lineage assignment through immunophenotyping depends on the expression levels of lineage-defining antigens for B lymphocytes, T lymphocytes, and myeloid cells. However, the WHO classification does not define the threshold for myeloperoxidase (MPO) positivity, which complicates the diagnosis of ALAL when it presents markers typical of acute lymphoblastic leukemia (ALL) but has MPO as the sole myeloid marker [2]. Moreover, the presence of small subclones or weak antigen expression further complicates the diagnosis. We present two cases of MPAL with challenging initial diagnoses, where lineage switching after the initial treatment required re-diagnosis and treatment modification.

This article was previously presented as a meeting abstract at the 86th Annual Meeting of the Japanese Society of Hematology on October 13, 2024.

## Case presentation

Case 1

A 53-year-old female presented with a fever of 39 °C, fatigue, and worsening systemic migratory arthralgia. She experienced progressively worsened dyspnea on mild exertion. Blood tests revealed severe anemia with a hemoglobin (Hb) level of 2.8 g/dL (normal range: 11.6-14.8 g/dL), elevated white blood cell (WBC) counts of 103.7 x 10^9^/L (normal range: 4.0-9.0 x 10^9^/L), including 92.5% abnormal blasts, 4% neutrophils, and 3.5% lymphocytes, along with a decreased platelet (PLT) count of 10 x 10^9^/L (normal range: 150-420 x 10^9^/L). The laboratory findings are summarized in Table 1.

**Table 1:** Laboratory findings of case 1

Parameters	Value	Normal range
Complete blood count		
White blood cells	103.7 x 10^9^/L	4.0-9.0 x 10^9^/L
Neutrophils	4.0%	40-70%
Lymphocytes	3.5%	15.0-60.0%
Monocytes	0.0%	0.0-10.0%
Eosinophils	0.0%	0.0-10.0%
Basophils	0.0%	0.0-5.0%
Blasts	92.5%	0.0%
Red blood cells	0.95×10^12^ /L	4.0-5.5×10^12^ /L
Hemoglobin	2.8 g/dL	11.6 -14.8 g/dL
Hematocrit	8.9%	40.7-50.1%
Mean corpuscular volume	93.7 fL	83.6-98.2 fL
Mean corpuscular hemoglobin concentration	31.5 g/dL	31.7-35.3 g/dL
Platelet	10×10^9^ /L	150 - 420 x 10^9^/L
Biochemistry		
Total protein	5.7 g/dL	6.6-8.1 g/dL
Albumin	2.8 g/dL	4.1-5.1 g/dL
Total bilirubin	0.5 mg/dL	0.4-1.5 mg/dL
Aspartate transaminase	29 U/L	13-30 U/L
Alanine transaminase	19 U/L	10-42 U/L
Lactate dehydrogenase	1196 U/L	124-222 U/L
Blood urea nitrogen	11 mg/dL	8-20 mg/dL
Uric acid	6.5 mg/dL	3.7-7.8 mg/dL
Creatinine	0.45 mg/dL	0.65-1.07 mg/dL
Sodium	129 mmol/L	138-145 mmol/L
Potassium	4.6 mmol/L	3.6-4.8 mmol/L
Chloride	96 mmol/L	101-108 mmol/L
C-reactive protein	11.10 mg/dL	0.00-0.14 mg/dL
Soluble interleukin-2 receptor	3810 IU/mL	157-474 IU/mL

**Figure 1:**
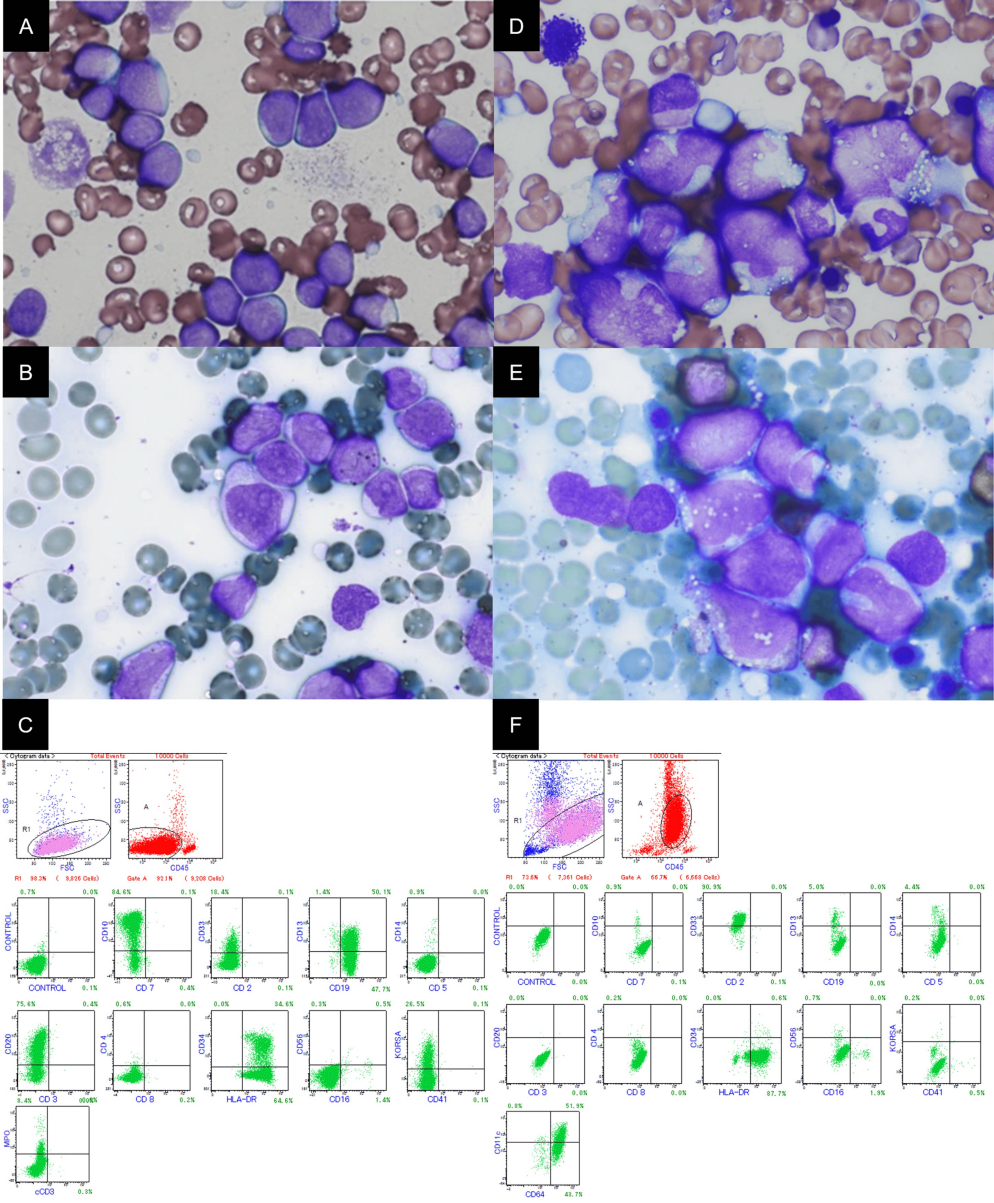
Bone marrow aspiration and flow cytometry of case 1 Bone marrow aspiration (May-Giemsa stain, peroxidase stain x 1000 magnification) and flow cytometry at first diagnosis of acute lymphoid leukemia (A-C) and relapse as acute myeloid leukemia phenotype (D-F)

Bone marrow examination revealed that 98% of the cells were small blast cells (around 15 μm) with a high nuclear-to-cytoplasmic ratio (approximately 90%) and pale and agranular cytoplasm. The nuclei were round with occasional clefts. The chromatin was coarse with one or two nucleoli. (Figure 1A). Additionally, the blast cells were negative for MPO staining (Figure 1B). Flow cytometric analysis (FCM) demonstrated that the majority of blasts were positive for CD10, CD19, CD20, CD13, CD33, and CD34. In contrast to the results obtained with MPO staining, a smaller population (8.4%) of blasts exhibited weak (dim) MPO expression as determined by FCM (Figure 1C). The conventional G-banding cytogenetic analysis revealed that the blast cells exhibited a complex set of chromosomal abnormalities: 46, XX, add (7) (p11.2), add (9) (p11), add (19) (p13), -20, and + mar1. [20/20]. Subsequently, the diagnosis of B-lymphoblastic leukemia/lymphoma was made according to the fifth edition of the WHO Classification [3]. She achieved hematological complete remission (CR) after induction therapy with rituximab combined with fractionated cyclophosphamide, vincristine, doxorubicin, and dexamethasone (R-hyper CVAD), alternating with high-dose methotrexate and cytarabine (MA) (rituximab 375 mg/m^2^, cyclophosphamide 350 mg/m^2^, vincristine 1.4 mg/m^2^, doxorubicin 50 mg/m^2^, dexamethasone 40 mg/body). The patient achieved molecular CR after subsequent consolidation treatments with MA and R-hyper CVAD.

Unfortunately, the disease recurred after the second cycle of MA. The blast cells were approximately 25 μm in size, exhibiting a nuclear-to-cytoplasmic ratio of approximately 70-90% and abundant cytoplasm with vacuoles. The nuclei were convoluted, with coarse chromatin and zero or a single nucleolus. In contrast to the initial diagnosis, the blasts now exhibited MPO positivity (Figures 1D, 1E). FCM analysis indicated that the blasts exhibited an immunophenotype consistent with acute myeloid leukemia (AML), showing positivity for CD11c, CD33, CD64, and HLA-DR, while being negative for CD10, CD19, and CD20 (Figure 1F). G-banding analysis revealed the blast cells exhibited a complex set of chromosomal abnormalities: 70, XX, -X, add (1) (p32) x3, +add (1) (q32), +3, add (7) (p11.2), +8, +8, add (9) (p11), -10, -20, -21, +mar1. [11/20] (including abnormal cells with common chromosomal abnormalities but not a complete match); 46, XX [9/20]. The chromosomal abnormalities evolved from those present at the initial diagnosis. Genetic testing of the leukemic cells revealed the presence of the Fms-like tyrosine kinase 3 internal tandem duplications (FLT3-ITD) mutation. Despite receiving re-induction therapy with azacitidine and venetoclax, along with salvage therapy using gilteritinib, the patient's condition has deteriorated.

Case 2

A 42-year-old male presented with inguinal lymphadenopathy. He had initially noticed left inguinal lymphadenopathy six weeks prior, followed by the development of right inguinal lymphadenopathy, with no subsequent improvement. He had been referred to the hematological unit with a suspicion of malignant lymphoma. Blood tests revealed a hemoglobin level of 10.8 g/dL, white blood cell counts of 2.0 x 10^9^/L with 13% blast cells, 6.5% neutrophils, 1% eosinophils, and 77% lymphocytes, along with platelet counts of 222 x 10^9^/L. The laboratory findings are summarized in Table 2.

**Table 2:** Laboratory findings of case 2

Parameters	Value	Normal range
Complete blood count		
White blood cells	2.0 x 10^9^/L	4.0-9.0 x 10^9^/L
Neutrophils	6.5%	40-70%
Lymphocytes	77%	15.0-60.0%
Monocytes	2.0%	0.0-10.0%
Eosinophils	1.0%	0.0-10.0%
Basophils	0.0%	0.0-5.0%
Metamyelocytes	0.5%	0.0%
Blasts	13.0%	0.0%
Red blood cells	2.85×10^12^ /L	4.0-5.5×10^12^ /L
Hemoglobin	10.8 g/dL	11.6 -14.8 g/dL
Hematocrit	31.2%	40.7-50.1%
Platelet	222×10^9^ /L	150 -420 x 10^9^/L
Biochemistry		
Total protein	7.5 g/dL	6.6-8.1 g/dL
Albumin	3.8 g/dL	4.1-5.1 g/dL
Total bilirubin	0.4 mg/dL	0.4-1.5 mg/dL
Aspartate transaminase	16 U/L	13-30 U/L
Alanine transaminase	21 U/L	10-42 U/L
Lactate dehydrogenase	178 U/L	124-222 U/L
Blood urea nitrogen	11 mg/dL	8-20 mg/dL
Uric acid	4.7 mg/dL	3.7-7.8 mg/dL
Creatinine	0.76 mg/dL	0.65-1.07 mg/dL
Sodium	135 mmol/L	138-145 mmol/L
Potassium	4.4 mmol/L	3.6-4.8 mmol/L
Chloride	107 mmol/L	101-108 mmol/L
C-reactive protein	1.98 mg/dL	0.00-0.14 mg/dL

**Figure 2:**
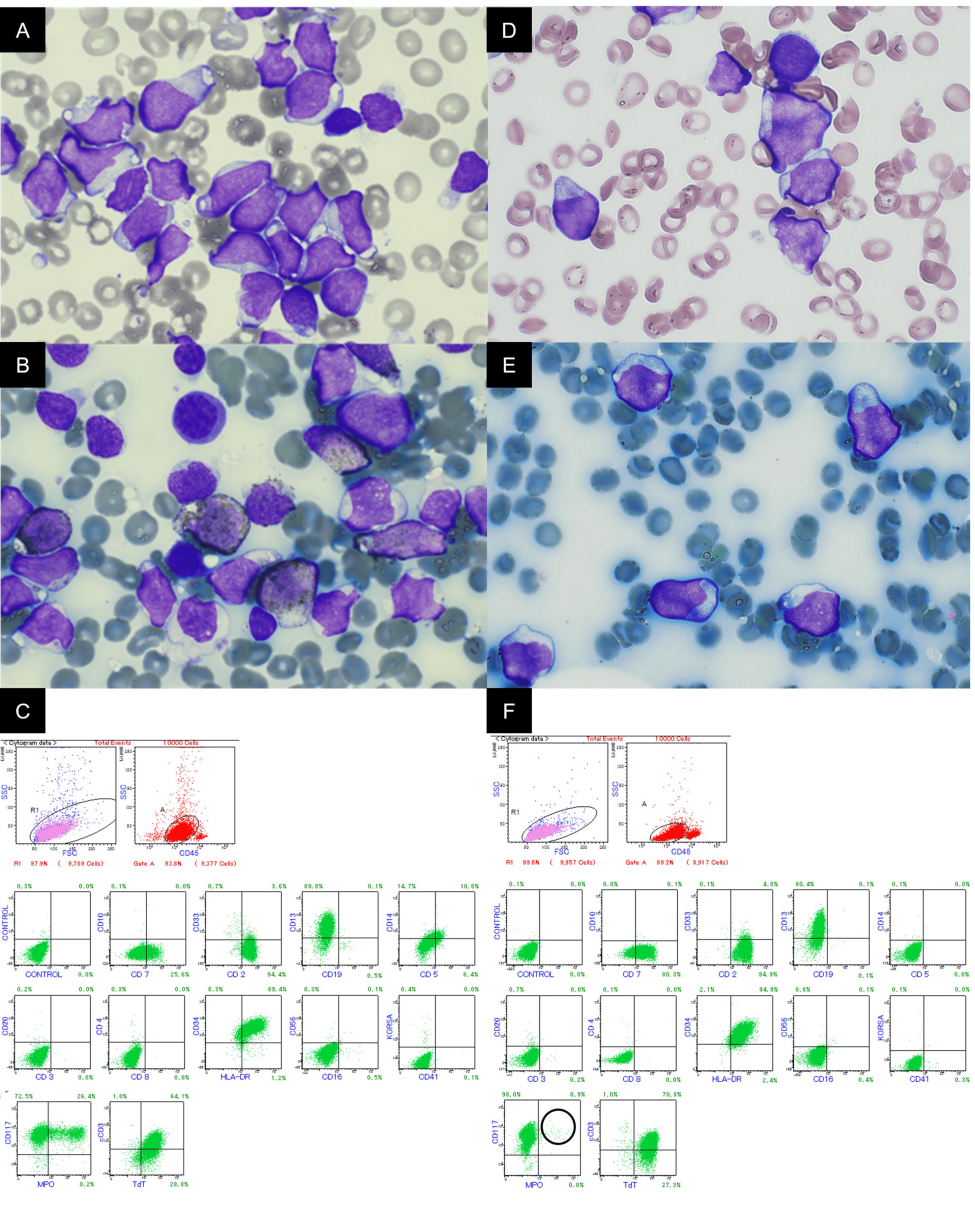
Bone marrow aspiration and flow cytometry of case 2 Bone marrow aspiration and flow cytometry at first diagnosis (A-C) and induction failure (D-F). A, D: May-Giemsa stain (x 1000 magnification). B, E: Peroxidase stain (x 1000 magnification). C, F: Flow cytometry. There were no significant morphological changes in Giemsa stain (D). Peroxidase stain and flow cytometry suggested that induction therapy with idarubicin and cytarabine had eliminated only myeloperoxidase-positive blasts (F; circle)

Bone marrow examination revealed 91.8% small blasts (approximately 20 μm in size) with a high nuclear-to-cytoplasmic ratio (70-95%) and pale cytoplasm. Some blasts displayed a distinctive “hand-mirror” morphology. The nuclei were irregular in shape, with fine chromatin and zero or one visible nucleoli. The blasts exhibited MPO positivity on IHC (Figures 2A, 2B). FCM identified two distinct populations of blasts: 65.1% tested positive for CD2, cytoplasmic CD3, CD13, CD117, HLA-DR, and TdT, whereas 26.6% expressed strong positivity for MPO (Figure 2C). The karyotype appeared normal through G-banding analysis. Southern blot hybridization demonstrated the presence of immunoglobulin (Ig) JH rearrangements; however, no rearrangements of the T-cell receptor (TCR) Jγ and Cβ1 genes were found.

A positive FLT3 point mutation was identified in the tyrosine kinase domain (FLT3-TKD). The patient was diagnosed with MPAL, T/myeloid. Induction therapy using idarubicin and cytarabine successfully eradicated MPO-positive blasts; however, blasts exhibiting T cell lineage markers remained (Figures 2D-2F). Subsequent treatment involved a regimen typically used for ALL, which included vincristine, prednisolone, L-asparaginase, daunorubicin, and cyclophosphamide (Japan Adult Leukemia Study Group 202O protocol), leading to hematological CR (hCR) [4]. The patient then underwent consolidation therapy with a high dose of Ara-C, followed by allogeneic hematopoietic stem cell transplantation (allo-HSCT). The conditioning regimen consisted of fludarabine and melphalan (140 mg/m²), combined with 4 Gy of total body irradiation (TBI).

## Discussion

MPAL is a rare entity, comprising only 2-5% of all acute leukemia cases [5]. In MPAL, patients with B/myeloid, T/myeloid, rare type, and trilineage phenotypes account for 58%, 36%, 4%, and 2%, respectively [6]. In MPAL, leukemic blast cells display a range of morphologies, resembling myeloblasts, monoblasts, and lymphoblasts, or may appear undifferentiated [6]. For the immunophenotype, the cutoff for MPO positivity ranges from 3% to 28% in FCM and is set at 3% in enzyme cytometry [7-9]. The WHO criteria for assigning myeloid lineage outline the strength of antigen expression but do not specify the percentage of blasts [2]. The International Consensus Classification also does not establish specific criteria or thresholds for MPO positivity [10]. However, it recommends exercising caution in cases where the MPO-positive population comprises fewer than 10% of the cells [10].

Case 1 was initially diagnosed as B-ALL, as the blasts exhibited morphological characteristics typical of ALL. A small subset (8.4%) showed weak MPO positivity, which did not provide sufficient evidence to classify this case as MPAL during the diagnostic process. Lineage shifts occur in approximately 6.7% of relapsed acute leukemia cases, frequently resulting from the development of small leukemic subclones under targeted treatment pressure [11]. These subclones may remain unnoticed during the initial diagnosis due to limitations in diagnostic sensitivity [12]. In this instance, the emergence of AML during relapse and a lineage shift indicated the presence of a previously unidentified myeloid subclones at the initial diagnosis. These subclones likely became the dominant population after the primary lymphoid cells were eliminated through ALL-like treatment.

Although this case did not fully meet the WHO criteria for MPAL due to the low MPO intensity, the clinical progression indicated it was likely MPAL, B/myeloid. Typically, MPO-positive blasts in B-ALL do not express other myeloid or monocyte markers, such as CD15, CD117, CD11b, and CD64, which differentiates them from MPAL, B/myeloid [13]. Conducting a more thorough FCM analysis at the initial diagnosis could have identified these markers and led to an earlier MPAL diagnosis. Thorough FCM analysis is essential to distinguish between B-ALL with isolated MPO positivity and MPAL. If B-ALL shows a small blast population with faint MPO expression, it is vital to ensure accurate classification to determine the best treatment strategy. Typically, the treatment plan is adjusted if the initial therapy is unsuccessful, favoring AML-like treatment over ALL-like treatment when the latter is ineffective [1]. We implemented this strategy; however, the growing subclones exhibited resistance to chemotherapy, which is commonly observed [10].

In Case 2, a regimen resembling AML began after the identification of MPO-positive blasts through cytochemical staining. However, after reviewing the FCM results following treatment initiation, a diagnosis of MPAL was established. The blasts displayed a distinctive “hand-mirror” morphology characterized by an elongated cytoplasmic tail extending from one pole (Figure 2A) [14]. This feature occurs more frequently in ALL than in AML [14,15]. These cells did not have typical open chromatin. Morphology by itself cannot clearly determine the immunophenotype; however, the concurrent identification of MPO-positive blasts requires careful consideration of MPAL. Due to the challenges in diagnosing MPAL, an accurate classification requires a thorough evaluation of both FCM results and blast morphology observed through May-Giemsa staining.

MPAL has a worse prognosis and outcomes compared to AML or ALL [16]. Its diversity complicates treatment choices, and no standard therapy is established [1]. While ALL-like induction therapy is preferred due to its higher CR rate, AML-like regimens have not shown superiority, even in cytochemically MPO-positive MPAL cases [17]. Genome-wide methylation analysis has allowed for the classification of MPAL into AML-type and ALL-type based on promoter CpG methylation patterns. This classification correlates with enhanced clinical responses when lineage-matched therapy is employed [18]. In Case 2, two distinct blast populations with different phenotypes were identified. The MPO-positive population was eradicated by 7+3 therapy, while the T cell phenotype population proved resistant. A subsequent ALL-like salvage therapy effectively eliminated the remaining blasts, resulting in hCR. This highlights the importance of accurately classifying MPAL to inform optimal treatment decisions. MPAL, especially the T/myeloid subtype, exhibits genetic similarities with early T cell precursor ALL (ETP-ALL) [19]. Asparaginase has been reported to yield positive outcomes in ETP-ALL [20], indicating its potential as a crucial therapy for MPAL, T/Myeloid. In Case 2, the use of salvage therapy with asparaginase proved beneficial, underscoring the significance of accurate classification in guiding treatment decisions.

In patients with MPAL, allo-HSCT is recommended during the initial CR when eligible, as it improves long-term outcomes for these patients [21]. A myeloablative conditioning regimen that incorporates total body irradiation has been reported to provide greater benefits [22]. In Philadelphia chromosome-negative ALL patients over 18 years who received pediatric-inspired intensive chemotherapy, molecular residual disease (MRD) status has been used to determine the need for allo-HSCT, allowing MRD-negative patients to potentially avoid transplantation [23]. However, MRD assessment in MPAL poses significant challenges due to its immunophenotypic heterogeneity and the potential for lineage switching or the emergence of subclones during treatment, as illustrated in Case 1. In Case 2, molecular MRD assessment through Ig or TCR rearrangement was complicated at the time of initial diagnosis. Unlike non-ETP ALL, the majority of ETP-ALL, a disease that exists between MPAL, T/myeloid, and T-ALL, typically lack TCR-γ gene rearrangements because the tumor cell of origin is immature, which further complicates MRD assessment [24]. The same challenges exist in MPAL, particularly in the T/myeloid subtype. Currently, there is no consensus on MRD-guided allo-HSCT strategies for MPAL, highlighting the need for further research. Advanced molecular techniques, such as next-generation sequencing, may be crucial for improving MRD evaluation in this condition.

## Conclusions

The multiphenotypic nature of MPAL presents significant challenges in both diagnosis and treatment-related decision-making. Diagnosing acute leukemia requires a close examination of potential subclones, ambiguous immunophenotypes, and irregular antigen expression. FCM is crucial for assessing subclones and the immunophenotype shift in leukemia. Furthermore, the clinical diversity of MPAL and its potential for subclone emergence require tailored treatment strategies that take into account immunophenotyping, cytogenetics, and molecular profiling. Progress in MRD assessments and molecular classification may also introduce new approaches to improve therapy and outcomes for MPAL patients.
